# Optical Coherence Tomography (OCT): A Brief Look at the Uses and Technological Evolution of Ophthalmology

**DOI:** 10.3390/medicina59122114

**Published:** 2023-12-03

**Authors:** Marco Zeppieri, Stefania Marsili, Ehimare Samuel Enaholo, Ayishetu Oshoke Shuaibu, Ngozi Uwagboe, Carlo Salati, Leopoldo Spadea, Mutali Musa

**Affiliations:** 1 Department of Ophthalmology, University Hospital of Udine, 33100 Udine, Italy; 2 Georgia Institute of Technology, School of Biological Sciences, Atlanta, GA 30332, USA; 3 Centre for Sight Africa, Nkpor, Onitsha 434109, Nigeria; 4 Africa Eye Laser Centre Ltd., Benin 300102, Nigeria; 5 Department of Optometry, University of Benin, Benin City 300238, Nigeria; 6 Eye Clinic, Policlinico Umberto I, “Sapienza” University of Rome, 00142 Rome, Italy

**Keywords:** imaging, optical coherence tomography (OCT), time-domain, spectral-domain, swept-source, ophthalmology, glaucoma, retinopathies

## Abstract

Medical imaging is the mainstay of clinical diagnosis and management. Optical coherence tomography (OCT) is a non-invasive imaging technology that has revolutionized the field of ophthalmology. Since its introduction, OCT has undergone significant improvements in image quality, speed, and resolution, making it an essential diagnostic tool for various ocular pathologies. OCT has not only improved the diagnosis and management of ocular diseases but has also found applications in other fields of medicine. In this manuscript, we provide a brief overview of the history of OCT, its current uses and diagnostic capabilities to assess the posterior segment of the eye, and the evolution of this technology from time-domain (TD) to spectral-domain (SD) and swept-source (SS). This brief review will also discuss the limitations, advantages, disadvantages, and future perspectives of this technology in the field of ophthalmology.

## 1. Introduction

The need for non-invasive medical investigations is critical to the rapid growth of the medical management of diseases, especially in delicate organs. Proper imaging eliminates the risk of trial-and-error medicine and the burden of invasive exploratory procedures before the commencement of treatment. In the eye, the visualization of ocular structures and physical investigations were limited to direct/indirect observation using technology such as ophthalmoscopy and tonometry, in addition to psychometric measures such as dark adaptation measurement, vergence tests, or information gleaned from the symptoms reported by the patient. The evolution of OCT has greatly revolutionized eye care. Anterior and posterior segment tissue can now be examined non-invasively, helping the clinician make faster and better management choices based on objective structural imaging [1]. Several structures of the eye can be quantitively assessed using this technology, including the cornea, iridocorneal angle, iris, retina, and optic nerve [2].

The aim of this minireview is to briefly assess the current literature regarding the characteristics, diagnostic capabilities, technological advances, limitations, advantages, and disadvantages of OCT for the assessment of the posterior segment structures of the eye. 

This overview of OCT technology is not intended to be a thorough and meticulous assessment of the current research in ophthalmology, but to briefly summarize the key concepts of OCT technology and the evolution of this diagnostic tool over time, as reported in the literature. This analysis can provide helpful information and reminders to clinicians in ophthalmology and in different fields about the diagnostic capabilities of OCT and can assist in the management of patients from a multidisciplinary approach.

## 2. Materials and Methods

A search of the related literature was conducted on the PubMed database using the keywords “Time Domain”, “Spectral Domain”, and “Swept Source Domain”, which yielded the following search criteria: ((“domain s”[All Fields] OR “domains”[All Fields] OR “protein domains”[MeSH Terms] OR (“protein”[All Fields] AND “domains”[All Fields]) OR “protein domains”[All Fields] OR “domain”[All Fields]) AND (“spectral”[All Fields] OR “spectrally”[All Fields]) AND (“domain s”[All Fields] OR “domains”[All Fields] OR “protein domains”[MeSH Terms] OR (“protein”[All Fields] AND “domains”[All Fields]) OR “protein domains”[All Fields] OR “domain”[All Fields]) AND “Swept”[All Fields] AND (“source”[All Fields] OR “sourced”[All Fields] OR “sources”[All Fields] OR “sourcing”[All Fields]) AND 2005/01/01:2023/12/31[Date-Publication]) AND (ffrft[Filter]). 

## 3. Results

**Figure 1:**
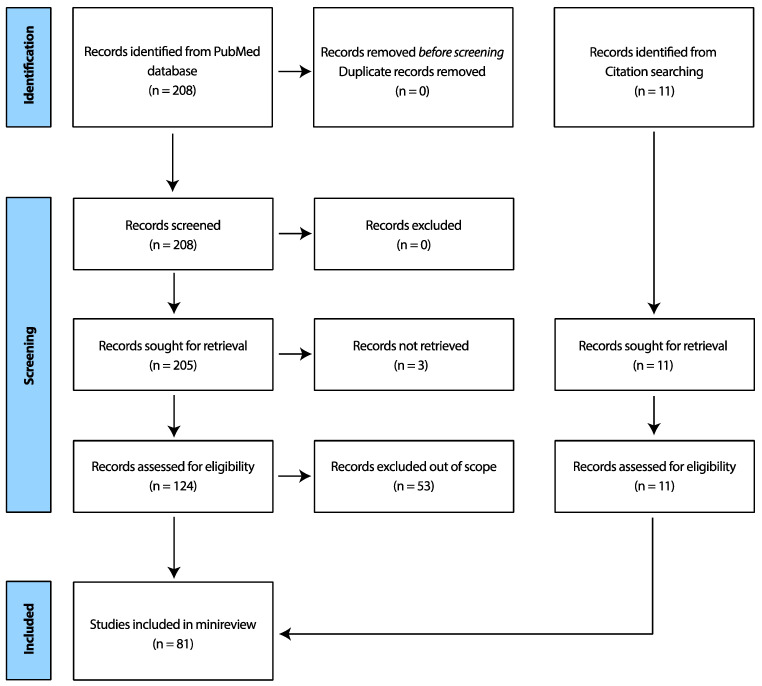
Prisma table showing search criteria process for this minireview.

Publications on OCT not relating directly to its use/evolution in ocular tissue were excluded. The authors acknowledge that this search string potentially may have unknowingly excluded important research papers. A Prisma table [3] is shown in Figure 1 below, delineating further sampling steps.

**Figure 2:**
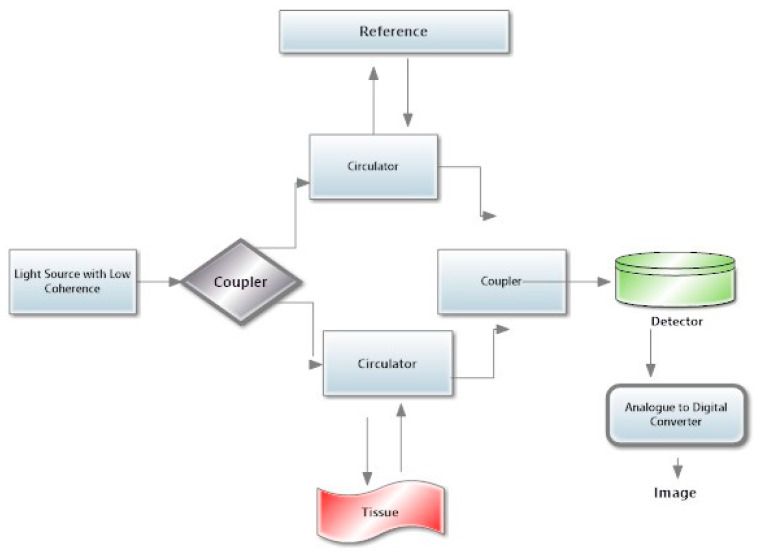
Schematic representation of OCT. The image shows the fundamental components and working principles of OCT. A low-coherence light source is a specialized light source emitting low-coherence light, typically in the near-infrared range, providing the necessary illumination for the imaging process. Couplers are used as optical components, such as beam splitters and fiber couplers, directing and dividing the light into the sample and reference arms. Optical circulators ensure unidirectional light propagation, guiding the sample light to the tissue and the reference light to the reference mirror. When using OCT to scan tissues, the emitted light interacts with the biological tissue, and the backscattered light carries information about the internal microstructure. High-sensitivity photodetectors are then used to capture the interference pattern formed by the combined sample and reference light, converting it into an electrical signal. Analog to Digital Converters (ADC) are part of OCT technology, in which the electrical signal is then fed into an Analog to Digital Converter, transforming the analogue signal into digital data for further processing. The digitized signals are processed to construct a high-resolution cross-sectional image of the tissue microstructure, revealing details such as layers and boundaries.

Before dwelling on the specific details of the various versions of this technology over the years, it is important to understand how to read the results provided by this technology. The different types of OCT technology are reported in Table 1. The general schematic representation of the instrumentation is shown in Figure 2. There are general steps to remember when interpreting an OCT printout of the posterior segment:

i. *Confirm the patient information:* patient information such as name, age, sex, and the date of the scan is usually printed on the top of the page.
ii. *Check the image quality:* Control the signal quality indicator (SQI) score on the printout. This will give you an idea of the quality of the image. High SQI scores indicate good image quality, while low SQI scores indicate lower quality.
iii. *Analyze the anatomy:* Look at the different layers of the retina and optic nerve head, which are normally labeled on the side of the printout. Make sure that the layers are clearly visible and identifiable.
iv. *Look at the thickness maps:* SD-OCT produces thickness maps that show the thickness of the retinal and optic nerve layers in different regions. These maps can help identify areas of thinning or thickening. Compare the thickness maps with the normative database to see if there are any areas of concern.
v. *Analyze the 3D images*: SD-OCT produces 3D images that show the retina and the optic nerve head in greater detail. Examine the 3D images to look for any abnormalities, such as swelling or distortion.
vi. *Review the measurement data*: SD-OCT provides detailed measurements of various retinal and optic nerve parameters. Review the data to look for any changes over time and compare the results with previous scans.
vii. *Consult a Specialist*: If you are unsure about how to interpret the SD-OCT printout, it is always best to consult an ophthalmologist or optometrist. They can help explain any unfamiliar terms or data and answer any questions you might have.

## 4. TD-OCT

TD-OCT is a medical imaging technology that creates detailed images of tissues in the body using near-infrared light. It is a two-dimensional imaging technology that measures light wave reflections from different depths within the tissue [13].

TD-OCT is one of the earliest forms of OCT. It was first described by Huang et al. [14]. The first TD-OCT system was based on the principles of low-coherence interferometry and utilized a Michelson interferometer [15]. In 1996, the first commercial TD-OCT system was released [16]. In the years following its initial development, TD-OCT was primarily employed for ophthalmic imaging, and many studies were conducted to evaluate its diagnostic and therapeutic potential [17]. The limitations of TD-OCT, such as slow image acquisition and limited axial resolution, led to the development of newer OCT technologies, such as Fourier Domain OCT (FD-OCT). Despite its limitations, TD-OCT remains an essential tool for various clinical applications, such as imaging of the anterior segment of the eye [18] and imaging through scattering media, where it may offer advantages over newer OCT technologies such as SD-OCT and SS-OCT [19].

Time-domain technology measures the time delay of a signal as it travels through a medium. TD-OCT involves sending a low-coherence light beam into a sample, such as biological tissue, and measuring the time it takes for the reflected light to return. The process begins with a low-coherence light source, which emits a beam of light split into two paths [20,21]. One path is reflected off a reference mirror, while the other is directed toward the sample. Later, the two light paths are recombined and reflected toward a detector, where their interference pattern is analyzed.

As the light travels through the sample, it is reflected by various structures within the tissue with different scattering properties. Some reflected light returns to the detector, interfering with the light reflected from the reference mirror. By measuring the time difference between the arrivals of the two light beams, the depth information of the structures within the tissue can be obtained.

The micron resolution limit refers to the smallest distance between two points that can be distinguished by an imaging system such as a microscope or a camera. The resolution limit is determined by several factors, including the wavelength of the light used, the lens’s numerical aperture, and the imaging system’s quality. The theoretical spatial resolution limit for an optical microscope is about 0.2 µm, which means that it can distinguish two points spaced at least 0.2 µm apart.

In TD-OCT, the resolution is limited by the spectral width of the light source used for imaging. Generally, the axial resolution of TD-OCT can reach 10–20 µm depending on the system’s specifications. This high resolution and the non-invasive nature of the modality make it well-suited for imaging structures such as cells, tissues, and organs [22]. To estimate the micro-resolution limit of a particular TD-OCT system, we can look at the system specifications to determine the wavelength and bandwidth of the light source and calculate the resolution using established equations or software tools. It is worth noting that light penetration may be affected by a range of factors, such as scattering and absorption in the biological tissue being imaged, as well as system noise and artifacts. These in turn limit the tissue imaging range and reduce the amplitude of the depth imaged in that tissue.

The TD-OCT printout contains a graph showing the reflected light intensity as a function of time or distance. Two peaks are usually depicted, the first peak representing the surface of the tissue being imaged, and the second peak representing the deeper structures within the tissue. The distance between the two peaks is known as the depth resolution, and it indicates the thickness of the tissue being imaged. The intensity of the reflected light at each point on the graph provides information about the optical properties of the tissue being imaged, such as its scattering and absorption coefficients. This information can be used to diagnose various medical conditions, including ophthalmic diseases. Interpreting the results from a TD-OCT printout requires a certain level of knowledge and expertise in medical imaging.

TD-OCT systems provide highly accurate and detailed images of biological tissues. Several independent manufacturers of TD-OCT systems include Thorlabs, Optovue, Heidelberg Engineering, NIDEK, Michelson Diagnostics, and Topcon.

The capabilities of TD-OCT include the following:

- *High-resolution imaging and low-coherence interferometry*: TD-OCT uses low-coherence interferometry that enables non-invasive optical imaging without damaging biological tissue. It gives high-resolution imagery with an axial resolution of up to 10–20 um, giving it an advantage over standard B-scans [23].
- *Depth imaging*: TD-OCT can produce cross-sectional images of biological tissue up to a depth of 2–3 mm with high resolution [24].
- *3D imaging*: TD-OCT can create 3D volumetric images of biological tissues by acquiring a series of 2D cross-sectional images [25].
- *Real-time imaging*: TD-OCT captures images in real-time, enabling the detection and monitoring of dynamic biological processes such as blood flow and tissue movement [12].
- *Versatility*: TD-OCT can image various biological tissues, including the eye, skin, and internal organs [24].

Overall, the capabilities of TD-OCT make it a useful imaging modality for research and clinical applications in areas such as ophthalmology, dermatology, and gastroenterology.

TD-OCT is not without its disadvantages. These include:

- *Limited imaging depth*: TD-OCT has a limited theoretical imaging depth of about 2–3 mm, which is further reduced when imaging deeper structures in the body.
- *Slow imaging speed*: The imaging speed of TD-OCT is relatively slow compared to newer technologies like SD-OCT and SS-OCT.
- *Motion artifacts*: Any motion during imaging can result in artifacts that can degrade image quality [26].

## 5. SD-OCT

The SD refers to an OCT imaging technology that uses a spectrometer to detect the interference pattern of the light reflected from different tissue layers. This allows for high-speed imaging of the tissue with high axial resolution. Compared to the previously used TD-OCT technology, SD-OCT provides faster image acquisition, higher resolution, and improved image quality [27]. SD-OCT is widely used in ophthalmology to diagnose and monitor various retinal diseases and other conditions affecting the eye.

SD-OCT was first introduced in the early 1990s as a variant of the original TD-OCT, which had limited imaging speed and resolution. SD-OCT’s development was made possible by the availability of broadband light sources and high-speed spectrometers, which allowed for faster and more accurate measurements of the optical reflections from tissues. In the early 2000s, commercial SD-OCT systems became available. They quickly gained popularity in ophthalmology, where they were used to image the retina and diagnose diseases such as macular degeneration, diabetic retinopathy, macular holes, vitreous-retinal traction, and glaucoma [1,4,5,6].

SD-OCT technology continues to evolve, improving image processing, motion correction, and 3D imaging capabilities. In recent years, SD-OCT has also found applications in other fields, such as cardiology, dermatology, and gastroenterology, where it is used to image the heart, skin, and gastrointestinal tract, respectively [28,29,30]. Overall, SD-OCT has revolutionized medical imaging by providing non-invasive, high-resolution images of biological tissues in real time. Its impact has been particularly significant in ophthalmology, where it has become the standard diagnostic tool for many eye diseases.

In SD-OCT, which uses Fourier-Domain OCT (FD-OCT), a broad spectrum of light is split into two arms; one is directed toward the tissue being imaged while the other arm serves as a reference. The reflected light from the tissue is then recombined with the reference light, and the resulting interference pattern is detected by a spectrometer [31,32]. SD-OCT works by sending a beam of light into a tissue and by measuring the reflections of the light that bounce back. The reflected light is then analyzed to create a high-resolution, cross-sectional image of the tissue. In simplified terms, the SD-OCT machine sends a beam of light through different tissue layers, e.g., the retina. The machine then uses advanced computer algorithms to process this information and create a highly detailed retina image [33].

The SD-OCT has resolution limits as precise as 1.3 um to 2.7 um [34,35]. The price range can vary depending on the device’s brand, model, and features.

Li et al. [36] suggested that the SD-OCT was the most diagnostic tool of the three available forms of OCT technology. Zhang et al. reported a good correlation between SD-OCT and TD-OCT [37]. It is also fast, gives an appreciably high resolution, and can be used to assess tissue in vivo non-invasively. Research has also shown the SD-OCT to be adequate in volumetric studies of the human cornea in vivo, showing the cornea cellular architecture and tear film [38]. As with other OCT technologies, the SD-OCT also has the familiar drawbacks of high cost, the need for specialist skills for operation, and false positives. False positives occur when artifacts are seen on the scan printout which do not have any clinical significance.

SD-OCT is continuously being developed to improve the technology and expand its scope of applications. Multimodal imaging, which combines SD-OCT with other imaging techniques like autofluorescence [39] and angiography [40], is now available and enables better diagnosis and management in a broader range of diseases, including age-related macular degeneration, diabetic retinopathy, and glaucoma. Improvements in machine learning algorithms and artificial intelligence could eventually automate the analysis of SD-OCT images, reducing the need for specialized training and expertise [41].

## 6. SS-OCT

Swept-Source OCT (SS-OCT) was introduced at the dawn of the second decade of the 21st century. Thus, it stands as a more novel imaging system in which further advancements were implemented to the capabilities of SD-OCT [41]. Similar to SD-OCT, the integral improvements in the system capabilities also leverage the application of FD-OCT technology to optimize higher-quality wavelength transduction within the frequency domain [42].

The essential optical components of SS-OCT machines include a sweeping tunable laser source and an intrinsic photoreceptor or photo-detector for receiving reflected light waves. Integrated tunable laser technology enables the use of long-wavelength emission (1040 to 1060 nanometers), which yields cross-sectional images in three-dimensional resolution, resulting in lower resolutions and deeper penetration. Like other ophthalmic OCT devices, posterior segment cross-sections are obtained as B-scan slices. Intrinsic extensive scanning laser interferometry facilitates a broader field of image acquisition [43]. High-frequency A-scan sweep rates, exceeding mean values of 100,000 Hertz (100 MHz), account for shorter test times and display acquisition faster than other optical coherence tomography modalities [44].

The improvements in OCT image resolution to about 11 um [45] also permit the detailed visualization of both superficial and deeper tissue layers: from the vitreoretinal interface and neurosensory retina to the choriocapillaris. Non-invasive SS-OCT Angiography (SS-OCTA) is generated via multiple B-scans acquired from single points around the retina [46] to create the phase-dependent imaging of static tissue against surrounding dynamic microvascular fluidics. SS-OCT devices provide greater sensitivity and a high signal-to-noise ratio [47]. Their systems are also capable of en-face imaging of the vitreoretinal interface, retina, and choroid [48]. This allows for the concurrent transverse visualization of superficial and deep retinal and choroidal pathology [49].

Mainstream manufacturers of SS-OCT systems include Topcon Medical Systems and Carl Zeiss Meditec. Some others include NIDEK, Heidelberg Engineering, Optovue, Santec Corporation, Thorlabs, Insight Photonic Solutions, and Excelitas Technologies. SS-OCT systems offer high-resolution imaging of biological tissues. The achievable micron resolution of SS-OCT depends on factors like the light source’s central wavelength and the system’s scanning speed. SS-OCT systems typically operate in near-infrared, with central wavelengths ranging from 1000 to 1300 nanometers [50]. The axial resolution of OCT is determined by the light source’s bandwidth, which can be calculated using the following formula: axial resolution ≈ λ^2/(2Δλ), where λ is the central wavelength and Δλ is the bandwidth [51].

In SS-OCT, the light source typically has a bandwidth in the tens to hundreds of nanometers range. As a result, the axial resolution of SS-OCT can range from a few micrometers to sub-micrometer resolution. It is essential to acknowledge that achieving the theoretical axial resolution limits in practical imaging situations can be challenging due to system noise, dispersion effects, and sample characteristics. Lateral resolution (transverse resolution) in OCT is determined by factors such as the focusing optics and beam profile, which can also vary based on the system setup and imaging conditions.

SS-OCT images can reveal various features and structures within the tissue. These include tissue layers, boundaries, blood vessels, cellular structures, and abnormalities. By examining the intensity and backscattering properties of the tissue, clinicians and researchers can identify and analyze these features. Interpreting SS-OCT images involves comparing the observed structures and components with known anatomical or pathological characteristics.

Clinicians and researchers use their expertise and knowledge to identify normal and abnormal tissue patterns, evaluate disease progression or treatment response, and make diagnoses or assessments. SS-OCT images can be further analyzed quantitatively to measure parameters such as tissue thickness, blood flow velocity, or reflectivity. This analysis can provide additional information for clinical decision-making or research purposes. In general, interpreting SS-OCT images requires specialized training and expertise. Clinicians, surgeons, and researchers working with SS-OCT must undergo specific training and retraining to correlate observed images with anatomical or pathological findings effectively.

The advantages of SS-OCT include the following:

- *Fixation compliance*: SS-OCT devices utilize long-wavelength infrared rays outside the visible light spectrum [38]. Hence, they improve patient comfort and subsequent compliance by minimizing the odds of generating motion artifacts.
- *Depth of ophthalmic penetration*: swept-source imaging modalities are also used to obtain enhanced depth imagery (EDI), with improvements upon SD EDI-OCT [49].
- *Reduced backscatter aberration*: the longer-wavelength optics employed in SS-OCT minimize backscatter and light absorption at the retinal pigment epithelium (RPE) layer.
- *Wide scanning ranges and wide imaging field*: sweeping tunable lasers in SS-OCT devices cover a wider field of view than other OCT devices when applied for both anterior segment and posterior segment imaging, as evidenced via the application of ultra-widefield swept-source OCT, (UWF) SS-OCT [52].
- *Improved signal strength limitations*: compared to SD-OCT, SS-OCT allows for better posterior segment image acquisition with a moderate decrease in ocular media transparency [11], e.g., with denser lens opacities and vitreous opacities.
- *High-quality en-face SS-OCT images*: this instrument eliminates side effects by reducing reliance on invasive indocyanine green angiography and fluorescein angiography [53].

The disadvantages of SS-OCT also include:

- *Higher cost margins*: The remarkable impact of SS-OCT on ophthalmic clinical research and the improved understanding of chorioretinal diseases notwithstanding, a major limitation to its adoption in routine patient care remains the exorbitant cost of its acquisition. Higher cost margins than SD-OCT are due to the adoption of lasers in SS-OCT systems.
- *Restricted availability*: an additional hindrance to the commercial mass production and adoption of SS-OCT has been because the technology offers the greatest value to medical retina specialty clinic settings and vitreoretinal surgical specialists (for preoperative evaluation and the longitudinal monitoring of treatment response).
- *Inherent paucity of normative databases*: Due to rare device availability, SS-OCT has limitations in glaucoma monitoring due to the limited sample size of normative data input on existing devices. It is also less difficult to find trials with machine learning algorithms in SD-OCT machines than SS-OCT [54].
- *Axial resolution drawbacks*: The enhanced deep choroid-sclera imaging capabilities of conventional SS-OCT limit its image resolution thresholds (compared to SD-OCT and TD-OCT), especially within the vitreous body and inner retinal layers [11]. These axial resolution limitations are due to the long coherence wavelengths and high emission frequency from super-luminescent diode lasers in SS-OCT systems. However, incorporating adaptive/assistive software has been explored to overcome this problem. Thus, future models might most probably overcome this issue [11].
- *SS-OCT also possesses diagnostic limitations*: in comparison with vascular endothelial-affinity dyes [46].

## 7. Comparative Uses of OCT Technology in Modern Clinical Ophthalmic Practice

TD-OCT is a good modality for imaging the retinal ganglion cell layer and other inner retinal layers with acceptable detail. TD-OCT is also capable of penetrating less-translucent media [55]. These features make it suitable for glaucoma screening [56], monitoring, and the differential diagnosis of other optic nerve diseases. TD-OCT is, however, prone to motion artifacts [57]. The limited depth of posterior segment penetration confounds its use for choroidal imagery [58].

On the other hand, SD-OCT provides more accurate anterior segment and retinal histological detail. For these properties, it is employed for intraoperative Anterior Segment Optical-OCT (AS-OCT)-guided visualization [59]. FD technology also enables improved axial resolution and en-face multimodal imaging comparable to invasive angiography [60]. These have further provided insights into inflammatory and vascular diseases of the retina and choroid, e.g., uveitis [61], neovascular AMD, and diabetic macular edema [62]. Improved images over TD-OCT also make it superior for optic nerve disease diagnosis and evaluation [63,64]. Consequently, SD-OCT is an essential tool for both corneal, vitreo-retinal, and glaucoma specialists as well [65].

SS-OCT is another suitable tool for imaging both the anterior and posterior intraocular segments. As a result of the cost-related inaccessibility of SS-OCT machines, their use in quantitative clinical decision-making can be limited. The paucity of quantitative values in SS-OCT systems means that they are, at times, more prone to false positives (red syndrome) and false negatives (green syndrome) in retinal ganglion cell layer thickness mapping. Green syndrome refers to the erroneous color coding given by the OCT printout. This is especially seen in cases when the analysis considers values as normal because they fall with the instrument’s normal, despite the presence of glaucoma or other diseases [36]. Red syndrome, however, is when the OCT codes analyze data as abnormal due to a contrast with normative values, despite an actual absence of disease [66]. Thus, SD-OCT is, for now, the more reliable glaucoma assessment tool [64]. Over time, improved data quantification in SS-OCT devices will most likely overcome this barrier. The enhanced horizontal 360° and axial scanning ranges of SS-OCT imaging already make it the top option for undertaking precision research, enveloping choroidal physiology into vascular disease [67] and myopia control [68].

**Figure 3:**
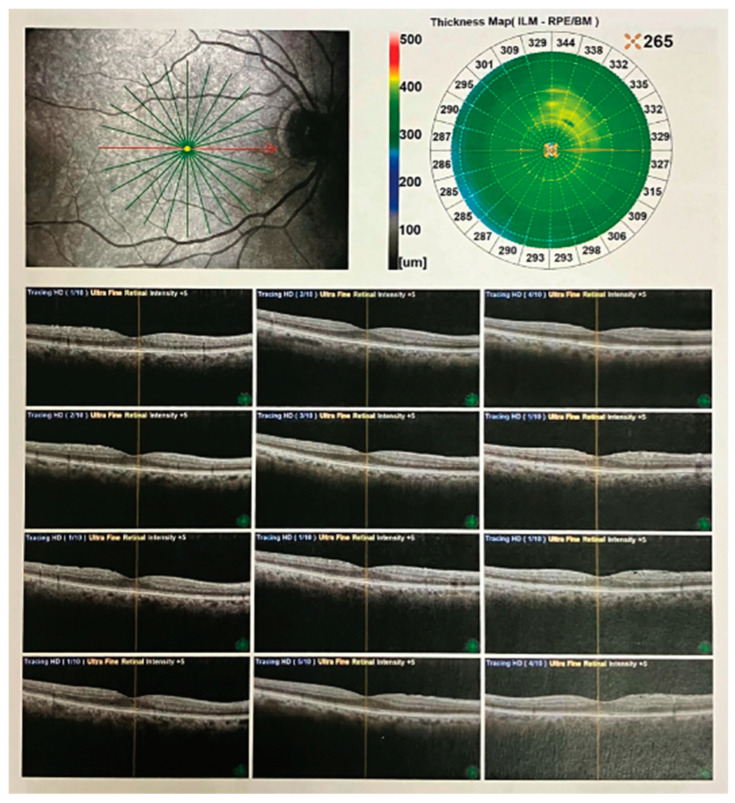
OCT radial scans of the macula in a patient with epiretinal membrane. SD-OCT images using a Nidek Optical Coherence Tomography (OCT) RS-3000 instrument. The OCT results show radial scans (indicated with the green lines) capturing the intricate microstructural details of the macula in a patient diagnosed with epiretinal membrane. The image displays a cross-sectional view of retinal layers (at the level of the macula indicated with the red line), showcasing the impact of the membrane on the retinal architecture. Prominently featured are disruptions in the inner retinal layers, including the macular fovea, and the distinct hyper-reflective band corresponding to the epiretinal membrane itself. This high-resolution OCT analysis provides valuable insights into the pathological changes associated with epiretinal membrane formation, aiding in the clinical assessment and management of retinal disorders.

**Figure 4:**
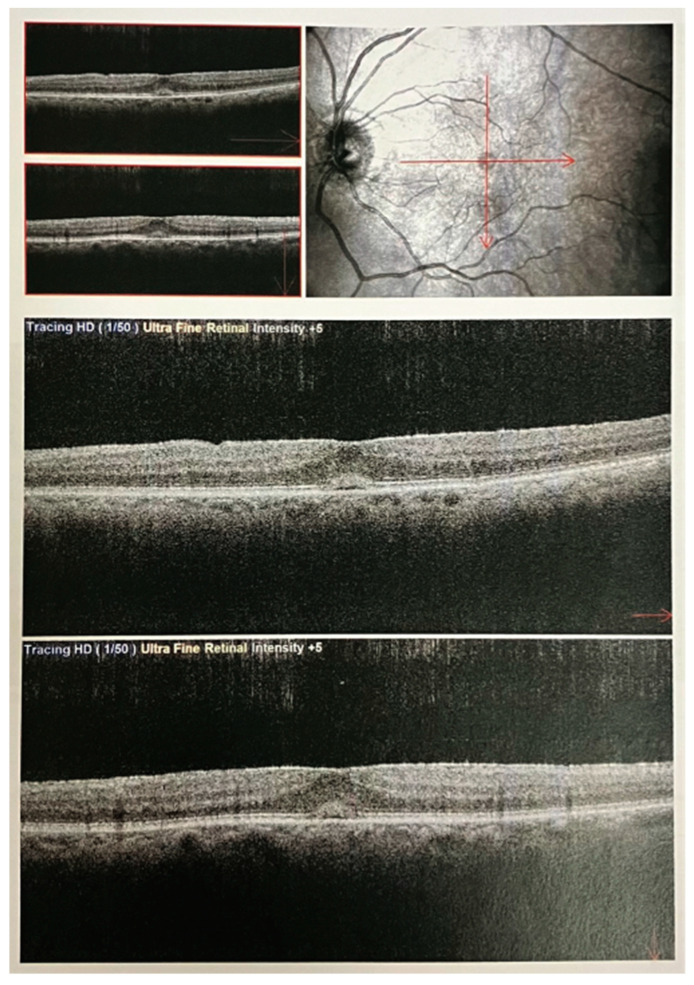
OCT macular scans in a patient with tractional pucker and linearization. SD-OCT images using a Nidek Optical Coherence Tomography (OCT) RS-3000 instrument. The OCT macular scans (at the specific position indicated with the red lines) illustrate the macular microstructure in a patient diagnosed with tractional pucker and linearization. The image captures detailed cross-sectional views of the macula, highlighting the pathological changes associated with tractional forces on the retinal surface. Notably, the scans reveal the presence of epiretinal membranes causing distortion and traction on the retinal layers, leading to the linearization of the macular architecture. The hyper-reflective bands represent the fibrous tissue causing the tractional forces, while disruptions in the normal retinal layers underscore the impact of this condition on retinal morphology.

**Figure 5:**
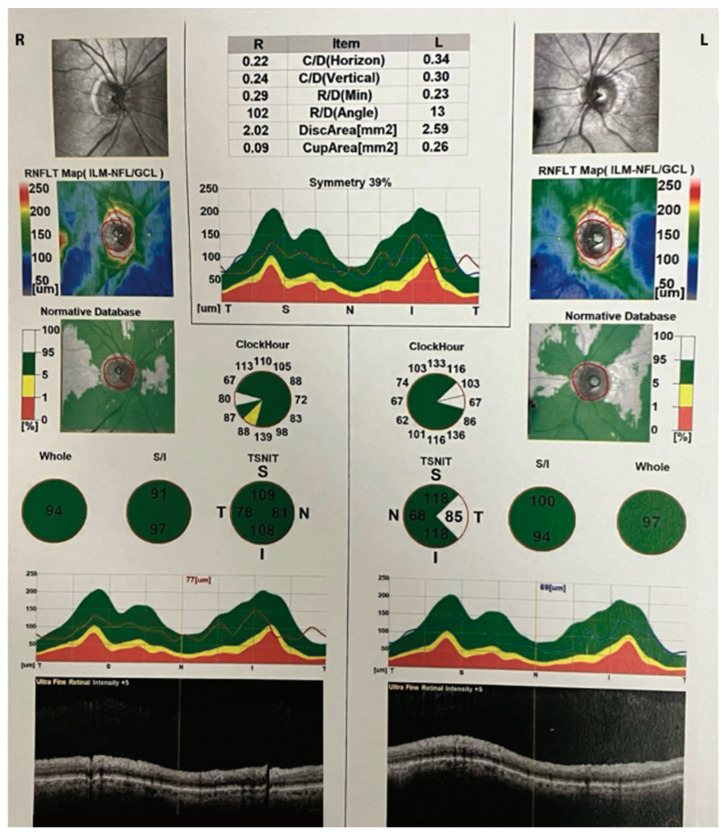
OCT scans showing retinal nerve fiber layer (RNFL) thickness values in clockwise sectors for both eyes. SD-OCT images using a Nidek Optical Coherence Tomography (OCT) RS-3000 instrument. The sections indicated in green are within normal limits compared with the normative age-related database. Yellow sections are considered borderline, while red is indicative of defective areas The OCT image depicts the peripapillary region, highlighting a subtle reduction in the thickness of the retinal nerve fiber layer (RNFL) in the inferior sector of the right eye, which is depicted in the yellow pie-shaped triangle above. The left eye is normal. The color-coded thickness map indicates a localized decrease in RNFL thickness, represented by the yellow, particularly in the inferior region surrounding the optic nerve head. This minor thinning of the peripapillary RNFL may suggest a subtle alteration in the nerve fiber layer density, which may have implications for visual function and requires close clinical monitoring. The precise mapping provided by OCT enables a detailed assessment of RNFL thickness, contributing valuable information to the early detection and management of subtle structural changes in the optic nerve head, which is important in patients with ocular hypertension and glaucoma.

Several clinical images of OCT have been included in the figures below (Figure 3, Figure 4 and Figure 5).

## 8. OCT Angiography (OCTA): A New Frontier in Ocular Imaging

Many ocular diseases are associated with vascular abnormalities. The conventional fluorescence angiography (FA) and indocyanine green angiography (ICGA) used for retinal and choroidal circulation assessment require the intravenous injection of contrast agents with potential side effects. Additionally, depth perception and detailed investigations of the retinal and choroidal networks are limited as FA and IGCA provide only two-dimensional images.

OCTA is a relatively new technology that allows the examiner to maximize the unique status of the eye, which is the only organ that permits the noninvasive in situ observation of the structure and function of its blood vessels. OCTA produces high-resolution, three-dimensional angiograms of the retinal and choroidal blood vessels. On the other hand, OCTA is not sensitive enough to detect vessel leakage and has a relatively small field of view. However, an understanding of the microvascular changes that occur in ocular diseases is key to the early diagnosis and monitoring of management therapies [69]. Some of the blood vessels in the retina and choroid are as small as 100 um [70] and standard visual examination is not sufficient to examine such tiny vessels.

The basic working principles of OCTA require repeated scans at the same location to detect motion, and it is based on the detection of contrast created by the movement of erythrocytes on a stationary background or static tissue. The derived OCTA signal relates to the flow rate of the red blood cells. However, overestimation of the flow signal due to bulk tissue and saccadic eye should be considered and motion correction applied to overcome artifacts. OCTA enables the observation of vascular adaptive processes to increase arterial blood flow such as vasospasm [71] and abnormal vasomotor tonus [72]. Kasahani et al. have indicated that indices including vessel density and the size of foveal avascular areas are key to grading microcirculation in the retinal blood vessels [73]. For example, an OCTA-observed reduction in vessel density has been reported in patients with poor hypertension control [74]. Hua et al. were also able to show that macula microvasculature changes can be assessed using OCTA even in non-hypertensive patients [75]. However, it should be noted that OCTA cannot measure vessel diameter as compared to SD-OCT, so it has its limitations [69]. The power of OCTA to visualize and quantify blood flow in the vasculature of the retina and choroid has enhanced the investigation of vascular physiology and pathology in ophthalmology. It provides information on flow-based anatomical and pathological changes that characterize ocular diseases such as, among others, neovascular and non-neovascular age-related macular degeneration, diabetic retinopathy, retinal vein and artery occlusions, inherited retinal dystrophies (IRDs), inflammatory diseases, glaucoma, and multiple sclerosis [73,74,75,76,77].

Although OCT has many advantages when compared to standard angiography procedures, the limitations of this technology must be considered. One of these is the small field of view. In addition, the high frequency of artifacts prevents a correct image interpretation. The most prevalent artifacts are those associated with eye motion, the misidentification of retinal layers, projections, and low optical coherence tomography; their identification and mitigation should be considered for the correct diagnosis and follow-up of patients [26].

## 9. OCT in Other Fields of Medicine

As OCT technology evolves in its capability and availability, its use in research and medical imaging will also increase, leading to better health decisions and outcomes [78,79]. Its fast, non-invasive, and detailed imagery makes it the diagnostic tool of choice in ophthalmology and other fields of medicine [80]. The high-resolution and non-invasive nature of OCT makes it a versatile tool across various medical specialties, providing valuable insights for diagnostic and guiding interventions. Cardiologists utilize intravascular OCT to image blood vessels, especially coronary arteries, thus aiding in the diagnosis and treatment of cardiovascular pathologies [81,82]. Dermatologists can use OCT to visualize skin layers in order to monitor wound healing, diagnose skin cancer, and assess skin structure [83]. OCT has also been shown to be useful in evaluating the gastrointestinal tract, aiding in diagnosing conditions like Barrett’s esophagus or inflammatory bowel diseases [84]. In respiratory medicine, OCT helps in detecting lung diseases and in guiding interventions like biopsies [85]. Oncology clinicians can find OCT helpful in detecting and monitoring the evolution of tumors, aiding in early diagnosis and treatment assessment [86]. With regard to neurology, OCT assists in imaging nerve tissues, helping in diagnosing conditions like multiple sclerosis [87]. OCT can be also used in dentistry for imaging teeth and oral tissues, aiding in diagnosis and treatment planning, especially for dental caries and periodontal diseases [88]. For ENT (Ear, Nose, Throat) specialists, OCT can be used for imaging the structure of the throat, nasal cavity, and ears, helping in the diagnosis of conditions such as vocal cord disorders and middle ear diseases [88].

## 10. Conclusions

The evolution of optical imaging is critical to the growth of investigative ophthalmology, enabling a better understanding of complex conditions including corneal diseases [76], retinal diseases, and glaucoma. Complex disease processes can now be studied as they unfold [77]. Despite the high costs of this technology and the requirement for specialized training and expertise, its advantages far outweigh its disadvantages. Its main strength consists of the real-time three-dimensional visualization of tissue structure and function without the necessity for a sample biopsy. High resolution, fast image acquisition, improved image quality, artifact reduction, and versatility have made OCT an innovative imaging device over time.

We hope that this manuscript can prove useful for all healthcare professionals who manage patients with different chronic pathologies, especially considering that the key to successful modern medicine is based on a multidisciplinary approach. An understanding of the guiding principles, advantages, and disadvantages serves as a springboard, driving the evolution of OCT. The development of better OCT technology to reach deeper tissues and acquire faster scans with fewer artifacts in real-time suggests promising times in clinical diagnostics, as seen in the evolution of blood flow monitoring in vivo [89]. From our perspective, machine learning algorithms and artificial intelligence could eventually automate the analysis of OCT images, reducing the need for specialized training and expertise.

## Figures and Tables

**Table 1:** Comparisons of the various OCT technologies.

	TD-OCT	SD-OCT	SS-OCT
1. Commercial Availability	1996 [4]	2006 [4]	2012 [4]
2. Principle of Operation	A beam splitter splits incident light into a reference wavelength which is adjusted for different depths while the sample light is reflected from the observed tissue and recombined at a sensor to give a single profile of the tissue [5].	Based on FD-OCT. It has similar principles to TD-OCT, but the sensor is upgraded to a spectrometer to split the reflected light into wavelengths (λ) using a diffractor. A combination of the individual images provided by the λs produces a Fourier transform to obtain depth information (A-scan) [6].	Also based on FD-OCT like SD-OCT. It possesses a sweeping tunable laser as its light source. A photoreceiver transduces the relayed signals into noise (images) [7].
3. Bandwidth	20 nm [8]	150 nm [8]	~1040–1080 nm [8]
4. Wavelength Frequency	810 nm [8]	840 nm [8]	~1050 nm [9]
5. Axial resolution	8–10 µm [9]	5–7 µm [9]	5.3 µm [10]
6. Scan Speeds	400 A-scans per second [11]	20,000–52,000 A-scans per second [11]	100,000–236,000 A-scans per second [11,12]

## Data Availability

No new data were created or analyzed in this study. Data sharing is not applicable to this article.
